# Chinese herbal medicine injections (CHMIs) for chronic pulmonary heart disease: A protocol for a Bayesian network meta-analysis

**DOI:** 10.1097/MD.0000000000024128

**Published:** 2021-01-22

**Authors:** Yuping Lei, Meili Wang, Guiqiang Sun, Yong Liu, Yapei Yang, Dong Hao

**Affiliations:** a Department of Cardiology, Liaocheng People's Hospital, Liaocheng; b Department of Cardiology, Jimo people's Hospital, Qingdao; c Department of Cardiology, Liaocheng Third People's Hospital, Liaocheng; d Department of Central Laboratory, Liaocheng People's Hospital, Liaocheng; e Department of Geriatrics, Liaocheng People's Hospital, Liaocheng, Shandong Province, PR China.

**Keywords:** Bayesian network meta-analysis, Chinese herbal medicine injections, chronic pulmonary heart disease, efficacy

## Abstract

**Background:**

Chinese herbal medicine injections (CHMIs) are frequently used for various refractory diseases including chronic pulmonary heart disease (CPHD). However, due to the diversity of CHMIs treatments, its relative effectiveness and safety remain unclear. In our study, Bayesian network meta-analysis will be used to identify differences in efficacy and safety between diverse CHMI for CPHD.

**Methods:**

Relevant randomized controlled trials (RCTs) and prospective controlled clinical trials published in PubMed, Google Scholar, Excerpt Medica Database, Medline, Cochrane Library, Web of Science, China Scientific Journal Database, China National Knowledge Infrastructure, Chinese Biomedical Literature Database and Wanfang Database will be systematic searched to identify eligible studies from their establishment to December 2020. The methodological qualities, including the risk of bias, will be evaluated using the Cochrane risk of bias assessment tool. Stata14.2 and WinBUGS 1.4.3 software were used for data synthesis. The evidentiary grade of the results will be also evaluated using the Grading of Recommendations Assessment, Development and Evaluation (GRADE) approach.

**Results:**

The results of this study will be published in a peer-reviewed journal, and provide reliable evidence for different CHMIs on CPHD.

**Conclusions:**

The findings will provide reference for evaluating the efficacy and safety of different CHMIs for CPHD, and provide a helpful evidence for clinicians to formulate the best adjuvant treatment strategy for CPHD patients.

**Trial registration number:**

INPLASY2020120004.

## 1 Introduction

Chronic pulmonary heart disease (CPHD) is a common heart disease, which has become an increasingly serious public problem that threatens peoples health and quality of life (QoL) around the world.^[1,2]^ Although the term “pulmonary heart disease” is popular in the medical literature, there is presently no consensual definition.^[2–6]^ World Health Organization defined CPHD as “hypertrophy of the right ventricle resulting from diseases affecting the function and/or structure of the lungs, and may further lead to heart failure”.^[1,7]^ Pulmonary hypertension caused by respiratory system disorders and/or chronic hypoxemia is the main pathological mechanisms of CPHD.^[1,2,8,9]^ Currently, the conventional treatment, including antibiotics, expectorants, antiasthmatic drugs, vasodilators, diuretics and antiarrhythic drugs etc. is the main clinical therapy for CPHD.^[1,2,10]^ However, it is universally acknowledged that long-time use of western medicine sometimes may cause drug resistance and toxic side effects, and therefore its clinical efficacy is still unsatisfactory.^[1–3]^ In view of these drawbacks to conventional therapy, there is a growing interest in the development of a new regimen with better tolerance and lower toxicity for patients with CPHD.

Many scholars indicated that the combination of Chinese and Western medicine for CPHD might be the potential trend of clinical treatment development in future.^[1,2,11–17]^ Chinese herbal medicine (CHM), as an essential component of complementary and alternative medicine, has gained more and more attention for CPHD.^[1,2,11–17]^ Chinese herbal medicine injection (CHMIs) are a new form of CHM preparation, which are prepared by extracting and purifying the effective and active compounds from herbs via modern scientific techniques and methods.^[18,19]^ It is guided by the theoretical system of syndrome differentiation of traditional Chinese medicine (TCM), and has the characteristics of rapid efficacy and convenient application.^[20]^ Clinical studies showed that many CHMIs could dilate blood vessels, reduce blood viscosity and platelet aggregation rate, improve blood circulation and promote the recovery of cardiac function.^[2,3,11,12]^ In spite of a growing number of studies on CHMIs for patients with CPHD in the late years, no comparison of efficacy between different CHMIs has been made.^[2,3,11,12]^ As a result, there is no decision-making conclusion as to which CHMI to choose in clinical practice. Considering that high-quality meta-analysis could provide reliable guidance for clinicians, the authors intend to complete a network meta-analysis based on Bayesian model.

### 1.1 Review question

Which CHMI is more effective for the treatment of patients with CPHD?

### 1.2 Objective

A Bayesian network meta-analysis will be performed to systematically evaluate the comparative effectiveness of different CHMIs for CPHD.

## 2 Methods

### 2.1 Protocol registration

Our protocol has been registered on the International Platform of Registered Systematic Review and Meta-Analysis Protocols (INPLASY). The registration number was INPLASY2020120004 (https://inplasy.com/inplasy-2020-12-0004/). The protocol of our meta-analysis will be reported according to Preferred Reporting Items for Systematic Review and Meta-Analysis Protocols (PRISMA-P) guidelines.^[21]^ Given that the meta-analysis is a secondary research which based on some previously published data, ethical approval is not necessary for our research.

### 2.2 Eligibility criteria

#### 2.2.1 Types of studies

Randomized controlled trials (RCTs) and quasi-RCTs or prospective controlled clinical trials that investigated the efficacy and safety of CHMIs for patients diagnosed with CPHD will be included in this systematic review. There will be no restrictions for blinding, population characteristics and duration of trials.

#### 2.2.2 Type of participants

Patients diagnosed with CPHD will be included in this study. No restrictions regarding age, gender, racial, region, education and economic status.

#### 2.2.3 Types of interventions

In the experimental group, CPHD patients must be treated with CHMI alone or in combination with other pharmacological interventions. One or more outcome measures, including the therapeutic effect, or hemorheology or blood gas indexes, or adverse events must be included in each study. There will be no restrictions with respect to dosage, duration, frequency, or follow-up time of treatment.

#### 2.2.4 Comparator

There will be no restrictions with respect to the type of comparator. The comparators are likely to include placebo, western medical therapies, supportive care, and other therapeutic methods.

#### 2.2.5 Exclusion criteria

Duplicated studies, papers without sufficient available data, clinical trials with inappropriate inclusion and exclusion criteria, non-comparative researches, case reports and series, meta-analysis, literature reviews, meeting abstracts, and other unrelated studies will be excluded from analysis.

#### 2.2.6 Type of outcome measurements

##### 2.2.6.1 Primary outcomes

1. Markedly effective rate (MER) and the total effective rate (TER);
2. QoL obtained from the corresponding scale;
3. Adverse events.

##### 2.2.6.2 Secondary outcomes

1. New York Heart Association (NYHA) classification;
2. Left ventricular ejection fraction (LVEF);
3. Mean pulmonary artery pressure (mPAP);
4. B-type natriuretic peptide (BNP);
5. Hemorrheology assessment, the hemorrheology index includes whole blood viscosity (WBV), plasma viscosity (PV), hematocrit, erythrocyte aggregation index (EAI) and content of fibrinogen (FBG);
6. Blood gas analysis, the blood gas indicator contains partial pressure of oxygen (PaO_2_), partial pressure of carbon dioxide (PaCO_2_), saturation of hemoglobin with oxygen (SaO_2_) and pH value.

### 2.3 Search strategy

To perform a comprehensive and focused search, experienced systematic review investigators will be invited to develop a search strategy. The plan searched terms are as follows: “pulmonary heart disease” or “chronic pulmonary heart disease” or “cor pulmonale” or “chronic cor pulmonale” or “fei yuan xing xin zang bing” or “fei xin bing” and “Chinese herbal medicine” or “Chinese herbal medicine preparation” or “Chinese herbal medicine injection” or “Chinese herbal injection” or “traditional Chinese medicine” or “traditional Chinese drug” or “Chinese herbal preparation” or “traditional Chinese preparation” or Chinese patent medicine” or “zhongcaoyao” or “CHMIs” or “TCM” et al. The preliminary retrieval strategy for PubMed is provided in Table 1, which will be adjusted in accordance with specific databases.

**Table 1:** Searching strategy in PubMed.

Search Strategy
#1. “pulmonary heart disease” or “chronic pulmonary heart disease” or “chronic cor pulmonale” or “cor pulmonale” or “PHD” or “CPHD” or “CP” or “CCP” [Title/Abstract].
#2. “cor pulmonale” [MeSH].
#3. #1 or #2.
#4. “Chinese herbal medicine” or “Chinese herbal medicine preparation” or “Chinese herbal medicine injection” or “Chinese herbal injection” or “traditional Chinese medicine” or “traditional Chinese drug” or “Chinese herbal preparation” or “traditional Chinese preparation” or Chinese patent medicine” or “CHMIs” or “TCM” [Title/Abstract].
#5. “Chinese medicine” or [MeSH].
#6. #4 or #5
#7. #3 and #6
#8. Limit #7 to “human”
#9. Limit #8 to “Clinical trial” [Publication Type]
#10. Limit #9 to yr = “-December 2020”

### 2.4 Information sources

Relevant RCTs, quasi-RCTs and high-quality prospective cohort studies will be systematically searched from PubMed, Google Scholar, Excerpt Medica Database, Medline, Cochrane Library, Web of Science, China Scientific Journal Database, China National Knowledge Infrastructure, Chinese Biomedical Literature Database and Wanfang Database from their inception to December 2020. Language is limited with English and Chinese.

### 2.5 Study selection and management

**Figure 1:**
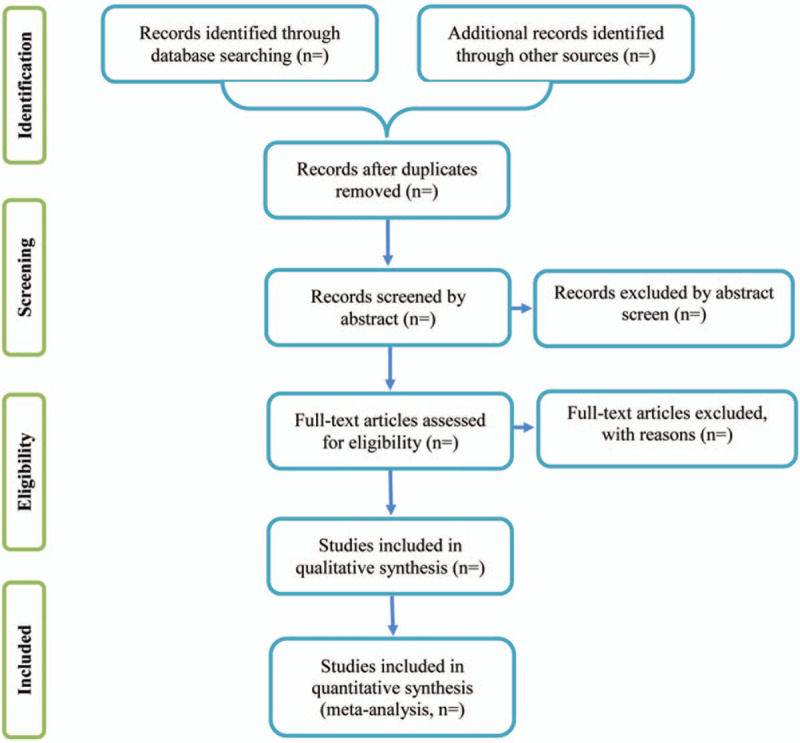
Study selection process for the meta-analysis.

First of all, all qualified documents will be extracted in the form of title and abstract, and preliminary screening will be conducted based on this information. On the basis of the previous step, the full text of the qualified literature will be obtained and further screened. All screening processes will be performed independently by the 2 authors (Yuping Lei and Meili Wang), and the reasons for each rejection will be documented. Disagreements between the 2 reviewers will be resolved by discussing with the third investigator (Guiqiang Sun). A PRISMA-compliant flow chart (Fig. 1) will be used to describe the selection process of eligible literatures. Endnote X7 software will be used for literature managing and records searching.

### 2.6 Data extraction and management

After screening the literature, the 2 authors (Yuping Lei and Meili Wang) will independently extract the information contained in the eligible literature to form a document feature table. The extracted data are as follows:

1. Study characteristics and methodology: country of study, the first authors name, year of publication, randomization, sample size, periods of data collection, follow-up duration, outcome measures, inclusion and exclusion criteria, et al.
2. Participant characteristics: age, gender, NYHA heart function classification, diagnostic criteria, et al.
3. Interventions: therapeutic means, dose, administration route, course of treatment, and duration of treatment, et al.
4. Outcome and other data: MER, TER, QoL, NYHA classification, LVEF, mPAP, BNP, hemorrheology indexes (WBV, PV, hematocrit, EAI and FBG), blood gas indicators (PaO_2_, PaCO_2_, SaO_2,_ and pH value), and adverse effects, et al.

### 2.7 Quality assessment

Two authors (Yuping Lei and Meili Wang) will independently assess risk of bias for each selected study in accordance with the Cochrane “Risk of bias” assessment tool which includes 7 items: random sequence generation, allocation concealment, blinding of participants and personnel, blinding of outcome assessment, incomplete outcome data, selective reporting and other bias.^[22,23]^ Each item will be evaluated at 3 levels: low risk, unclear, and high risk. Effective Practice and Organisation of Care (EPOC) guidelines will be used to assess the risks of non-RCTs.^[24]^ Any disagreements will be resolved via discussion with a third researcher (Guiqiang Sun).

### 2.8 Statistical analysis

We will conduct a conventional pairwise meta-analysis of the direct comparison results obtained from the literature. Continuous data will be presented as mean difference (MD) or standardized mean difference (SMD) with their confidence intervals (CIs). Dichotomous data will be recorded as odds ratio with 95% CIs. Stata 14.2 (Stata Corp., College Station, TX, USA) and WinBUGS1.4.3 (MRC Biostatistics Unit, Cambridge, UK) through the GeMTC package will be used to perform network meta-analysis to synthesize direct and indirect evidence. The network meta-analysis will be undertaken primarily in WinBUGS using the Markov chain Monte Carlo method.^[25]^ Convergence of the simu-lations will be evaluated with potential scale reduction factor and Gelman-Rubin-Brooks plots.^[26]^ The selection of the final model will depend on the deviance information criterion value. Generally, a model with a smaller deviance information criterion value is better.^[27,28]^ We will calculate the ranking probabilities for all treatments of being at each possible rank for each intervention, using the surface under the cumulative ranking curve (SUCRA), where the SUCRA values can range from zero to one. The evidence relationship of included studies will be figured out by Stata software. If there is a “closed loop,” the node splitting method will be used to evaluate the inconsistency of each loop.^[28–30]^

### 2.9 Assessment of heterogeneity

The heterogeneity of each pairwise comparison will be tested by χ^2^ statistics and the *I*^2^ statistics.^[31]^ When the *P* value was >.1, and *I*^*2*^ was <50%, it suggested that there was no statistical heterogeneity and the Mantel-Haenszel fixed-effects model was used for meta-analysis. Otherwise, a random-effects mode will be used to calculate the outcomes.

### 2.10 Subgroup and meta-regression analysis

If the χ^2^ and *I*^*2*^ test detect obvious heterogeneity between studies, we will explore sources of heterogeneity with respect to age, region, treatment duration and types of CHMI by subgroup analysis and meta-regression.

### 2.11 Sensitivity analysis

Sensitivity analysis will be conducted to assess the reliability and robustness of the aggregation results via eliminating trials with low-quality. A summary table will report the results of the sensitivity analyses.

### 2.12 Publication bias analysis

Funnel plot will be performed to analyze the existence of publication bias if the included studies are sufficient (n ≥ 10). If the funnel chart has poor symmetry, it indicates publication bias.^[32]^

### 2.13 Assess the quality of evidence

The evidence grade will be assessed by using the guidelines of the Grading of Recommendations, Assessment, Development, and Evaluation (GRADE, https://gradepro.org/). The quality of all evidence will be assessed at 4 levels: high, moderate, low, and very low.^[33]^

### 2.14 Dissemination plans

The results of this study will be published in a peer-reviewed journal, and provide reliable evidence for different CHMIs on CPHD.

## 3 Discussion

TCM is one of the world overall medical systems, and is considered to be promising to improve various refractory diseases such as CPHD, malaria and 2019 novel coronavirus, etc.^[11,12,34–36]^ Up to now, although some traditional meta-analysis suggested that CHMIs were effective in the treatment of CPHD, but there was still no comparison on the therapeutic effect between different CHMIs.^[2,3,11,12]^ Conventional meta-analysis cannot solve this problem well. Compared with the double-arm meta-analysis, network meta-analysis can synthesize multiple correlation factors and direct or indirect comparisons simultaneously by summarizing different interventions for the same disease.^[18]^ Moreover, it can provide evidence for identifying optimal therapies based on the rankings of different outcomes.^[18]^ In this study, the author plans to rank the efficacy and safety of difference CHMIs for CPHD through direct or indirect comparison. We hope that the study results will help to figure out which 1 or which combination of these interventions has the relatively optimal effect and safety and provide decision-making reference for clinicians, patients, and policy-makers to a certain extent.

There are some limitations that may affect the drawn conclusion. There may be a language bias with the limitation of English and Chinese studies. In addition, some conclusions drawn from this meta-analysis may have a certain degree of heterogeneity due to different CHMIs, NYHA score and treatment duration among included trials.

## Author contributions

**Conceptualization:** Yuping Lei, Dong Hao.

**Data curation:** Yuping Lei, Meili Wang, Guiqiang Sun.

**Formal analysis:** Yuping Lei, Meili Wang, Yong Liu.

**Funding acquisition:** Yapei Yang.

**Investigation:** Yuping Lei, Meili Wang, Guiqiang Sun, Yong Liu.

**Methodology:** Yuping Lei, Meili Wang, Yapei Yang.

**Project administration:** Dong Hao.

**Resources:** Yuping Lei, Dong Hao.

**Software:** Yuping Lei, Dong Hao.

**Supervision:** Yuping Lei, Dong Hao.

**Validation:** Yong Liu, Yapei Yang, Dong Hao.

**Visualization:** Yuping Lei, Meili Wang, Guiqiang Sun.

**Writing – original draft:** Yuping Lei, Meili Wang, Guiqiang Sun, Yong Liu.

**Writing – review & editing:** Yapei Yang, Dong Hao.
